# Genetically Determined Height and Risk of Non-hodgkin Lymphoma

**DOI:** 10.3389/fonc.2019.01539

**Published:** 2020-01-28

**Authors:** Amy Moore, Eleanor Kane, Zhaoming Wang, Orestis A. Panagiotou, Lauren R. Teras, Alain Monnereau, Nicole Wong Doo, Mitchell J. Machiela, Christine F. Skibola, Susan L. Slager, Gilles Salles, Nicola J. Camp, Paige M. Bracci, Alexandra Nieters, Roel C. H. Vermeulen, Joseph Vijai, Karin E. Smedby, Yawei Zhang, Claire M. Vajdic, Wendy Cozen, John J. Spinelli, Henrik Hjalgrim, Graham G. Giles, Brian K. Link, Jacqueline Clavel, Alan A. Arslan, Mark P. Purdue, Lesley F. Tinker, Demetrius Albanes, Giovanni M. Ferri, Thomas M. Habermann, Hans-Olov Adami, Nikolaus Becker, Yolanda Benavente, Simonetta Bisanzi, Paolo Boffetta, Paul Brennan, Angela R. Brooks-Wilson, Federico Canzian, Lucia Conde, David G. Cox, Karen Curtin, Lenka Foretova, Susan M. Gapstur, Hervé Ghesquières, Martha Glenn, Bengt Glimelius, Rebecca D. Jackson, Qing Lan, Mark Liebow, Marc Maynadie, James McKay, Mads Melbye, Lucia Miligi, Roger L. Milne, Thierry J. Molina, Lindsay M. Morton, Kari E. North, Kenneth Offit, Marina Padoan, Alpa V. Patel, Sara Piro, Vignesh Ravichandran, Elio Riboli, Silvia de Sanjose, Richard K. Severson, Melissa C. Southey, Anthony Staines, Carolyn Stewart, Ruth C. Travis, Elisabete Weiderpass, Stephanie Weinstein, Tongzhang Zheng, Stephen J. Chanock, Nilanjan Chatterjee, Nathaniel Rothman, Brenda M. Birmann, James R. Cerhan, Sonja I. Berndt

**Affiliations:** ^1^Division of Cancer Epidemiology and Genetics, National Cancer Institute, Bethesda, MD, United States; ^2^Department of Health Sciences, University of York, York, United Kingdom; ^3^Department of Computational Biology, St. Jude Children's Research Hospital, Memphis, TN, United States; ^4^Laboratory of Translational Genomics, Division of Cancer Epidemiology and Genetics, National Cancer Institute, Bethesda, MD, United States; ^5^Department of Health Services, Policy, and Practice, Brown University School of Public Health, Providence, RI, United States; ^6^Center for Gerontology and Healthcare Research, Brown University School of Public Health, Providence, RI, United States; ^7^Epidemiology Research Program, American Cancer Society, Atlanta, GA, United States; ^8^Epidemiology of Childhood and Adolescent Cancers Group, Inserm, Center of Research in Epidemiology and Statistics Sorbonne Paris Cité (CRESS), Paris, France; ^9^Université Paris Descartes, Paris, France; ^10^Registre des hémopathies malignes de la Gironde, Institut Bergonié, Bordeaux, France; ^11^Cancer Epidemiology and Intelligence Division, Cancer Council Victoria, Melbourne, VIC, Australia; ^12^Department of Hematology and Medical Oncology, Emory University School of Medicine, Atlanta, GA, United States; ^13^Department of Health Sciences Research, Mayo Clinic, Rochester, MN, United States; ^14^Department of Hematology, Hospices Civils de Lyon, Lyon, France; ^15^Department of Hematology, Université Lyon-1, Lyon, France; ^16^Equipe Experimental and Clinical Models of Lymphomagenesis, Cancer Research Center of Lyon, Institut National de Santé et de la Recherche Médicale UMR1052 Pierre Benite, Lyon, France; ^17^Division of Hematology and Hematologic Malignancies, Department of Internal Medicine and Huntsman Cancer Institute, University of Utah School of Medicine, Salt Lake City, UT, United States; ^18^Department of Epidemiology and Biostatistics, University of California, San Francisco, San Francisco, CA, United States; ^19^Center for Chronic Immunodeficiency, University Medical Center Freiburg, Freiburg im Breisgau, Germany; ^20^Division of Environmental Epidemiology, Institute for Risk Assessment Sciences, Utrecht University, Utrecht, Netherlands; ^21^Julius Center for Health Sciences and Primary Care, University Medical Center Utrecht, Utrecht, Netherlands; ^22^Clinical Genetics Service, Department of Medicine, Memorial Sloan Kettering Cancer Center, New York, NY, United States; ^23^Department of Medicine, Solna, Karolinska Institutet, Stockholm, Sweden; ^24^Hematology Center, Karolinska University Hospital, Stockholm, Sweden; ^25^Department of Environmental Health Sciences, Yale School of Public Health, New Haven, CT, United States; ^26^Centre for Big Data Research in Health, University of New South Wales, Sydney, NSW, Australia; ^27^Department of Preventive Medicine, USC Keck School of Medicine, University of Southern California, Los Angeles, CA, United States; ^28^Norris Comprehensive Cancer Center, USC Keck School of Medicine, University of Southern California, Los Angeles, CA, United States; ^29^Cancer Control Research, BC Cancer, Vancouver, BC, Canada; ^30^School of Population and Public Health, University of British Columbia, Vancouver, BC, Canada; ^31^Division of Health Surveillance and Research, Department of Epidemiology Research, Statens Serum Institut, Copenhagen, Denmark; ^32^Centre for Epidemiology and Biostatistics, Melbourne School of Population and Global Health, University of Melbourne, Melbourne, VIC, Australia; ^33^Department of Internal Medicine, Carver College of Medicine, The University of Iowa, Iowa City, IA, United States; ^34^Department of Obstetrics and Gynecology, New York University School of Medicine, New York, NY, United States; ^35^Department of Environmental Medicine, New York University School of Medicine, New York, NY, United States; ^36^Perlmutter Cancer Center, NYU Langone Medical Center, New York, NY, United States; ^37^Ontario Health Study, Toronto, ON, Canada; ^38^Division of Public Health Sciences, Fred Hutchinson Cancer Research Center, Seattle, WA, United States; ^39^Interdisciplinary Department of Medicine, University of Bari, Bari, Italy; ^40^Division of General Internal Medicine, Mayo Clinic College of Medicine and Science, Rochester, MN, United States; ^41^Department of Medical Epidemiology and Biostatistics, Karolinska Institutet, Stockholm, Sweden; ^42^Department of Epidemiology, Harvard School of Public Health, Boston, MA, United States; ^43^Division of Cancer Epidemiology, German Cancer Research Center, Heidelberg, Germany; ^44^Cancer Epidemiology Research Programme, Catalan Institute of Oncology-IDIBELL, L'Hospitalet de Llobregat, Barcelona, Spain; ^45^CIBER de Epidemiología y Salud Pública, Barcelona, Spain; ^46^Regional Cancer Prevention Laboratory, Oncological Network, Prevention and Research Institute (ISPRO), Florence, Italy; ^47^The Tisch Cancer Institute, Icahn School of Medicine at Mount Sinai, New York, NY, United States; ^48^International Agency for Research on Cancer (IARC), Lyon, France; ^49^Genome Sciences Centre, BC Cancer, Vancouver, BC, Canada; ^50^Department of Biomedical Physiology and Kinesiology, Simon Fraser University, Burnaby, BC, Canada; ^51^Genomic Epidemiology Group, German Cancer Research Center, Heidelberg, Germany; ^52^Bill Lyons Informatics Centre, UCL Cancer Institute, University College London, London, United Kingdom; ^53^INSERM U1052, Cancer Research Center of Lyon, Centre Léon Bérard, Lyon, France; ^54^Department of Internal Medicine, University of Utah School of Medicine, Salt Lake City, UT, United States; ^55^Department of Cancer Epidemiology and Genetics, Masaryk Memorial Cancer Institute and MF MU, Brno, Czechia; ^56^Department of Hematology, Centre Léon Bérard, Lyon, France; ^57^Department of Internal Medicine, Huntsman Cancer Institute, Salt Lake City, UT, United States; ^58^Department of Immunology, Genetics and Pathology, Uppsala University, Uppsala, Sweden; ^59^Division of Endocrinology, Diabetes and Metabolism, The Ohio State University, Columbus, OH, United States; ^60^INSERM U1231, Registre des Hémopathies Malignes de Côte d'Or, University of Burgundy and Dijon University Hospital, Dijon, France; ^61^Department of Medicine, Stanford University School of Medicine, Stanford, CA, United States; ^62^Environmental and Occupational Epidemiology Unit, Oncological Network, Prevention and Research Institute (ISPRO), Florence, Italy; ^63^Department of Pathology, AP-HP, Necker Enfants malades, Université Paris Descartes, Sorbonne Paris Cité, Paris, France; ^64^Department of Epidemiology, University of North Carolina at Chapel Hill, Chapel Hill, NC, United States; ^65^Carolina Center for Genome Sciences, University of North Carolina at Chapel Hill, Chapel Hill, NC, United States; ^66^CPO-Piemonte and Unit of Medical Statistics and Epidemiology, Department Translational Medicine, University of Piemonte Orientale, Novara, Italy; ^67^School of Public Health, Imperial College London, London, United Kingdom; ^68^Department of Family Medicine and Public Health Sciences, Wayne State University, Detroit, MI, United States; ^69^Genetic Epidemiology Laboratory, Department of Pathology, University of Melbourne, Melbourne, VIC, Australia; ^70^School of Nursing and Human Sciences, Dublin City University, Dublin, Ireland; ^71^Cancer Epidemiology Unit, University of Oxford, Oxford, United Kingdom; ^72^International Agency for Research on Cancer, World Health Organization, Lyon, France; ^73^Department of Epidemiology, Brown School of Public Health, Providence, RI, United States; ^74^Department of Biostatistics, Bloomberg School of Public Health, Johns Hopkins University, Baltimore, MD, United States; ^75^Department of Oncology, School of Medicine, Johns Hopkins University, Baltimore, MD, United States; ^76^Channing Division of Network Medicine, Department of Medicine, Brigham and Women's Hospital and Harvard Medical School, Boston, MA, United States

**Keywords:** non-Hodgkin lymphoma, height, genetics, chronic lymphocytic leukemia, diffuse large B-cell lymphoma, follicular lymphoma, marginal zone lymphoma, polygenic risk score

## Abstract

Although the evidence is not consistent, epidemiologic studies have suggested that taller adult height may be associated with an increased risk of some non-Hodgkin lymphoma (NHL) subtypes. Height is largely determined by genetic factors, but how these genetic factors may contribute to NHL risk is unknown. We investigated the relationship between genetic determinants of height and NHL risk using data from eight genome-wide association studies (GWAS) comprising 10,629 NHL cases, including 3,857 diffuse large B-cell lymphoma (DLBCL), 2,847 follicular lymphoma (FL), 3,100 chronic lymphocytic leukemia (CLL), and 825 marginal zone lymphoma (MZL) cases, and 9,505 controls of European ancestry. We evaluated genetically predicted height by constructing polygenic risk scores using 833 height-associated SNPs. We used logistic regression to estimate odds ratios (OR) and 95% confidence intervals (CI) for association between genetically determined height and the risk of four NHL subtypes in each GWAS and then used fixed-effect meta-analysis to combine subtype results across studies. We found suggestive evidence between taller genetically determined height and increased CLL risk (OR = 1.08, 95% CI = 1.00–1.17, *p* = 0.049), which was slightly stronger among women (OR = 1.15, 95% CI: 1.01–1.31, *p* = 0.036). No significant associations were observed with DLBCL, FL, or MZL. Our findings suggest that there may be some shared genetic factors between CLL and height, but other endogenous or environmental factors may underlie reported epidemiologic height associations with other subtypes.

## Introduction

In epidemiologic studies, taller adult height has been associated with an increased risk of several subtypes of non-Hodgkin lymphoma (NHL) (1); however, the results have not been consistent across studies for common subtypes, such as diffuse large B-cell lymphoma (DLBCL), follicular lymphoma (FL), and chronic lymphocytic leukemia/small cell lymphoma (CLL) (2–13). Taller height has also been positively associated with other cancers in both epidemiologic and Mendelian randomization studies (14–16). The underlying reason or biological mechanism for these associations is not understood. Adult attained height is not thought to be causally related to cancer, but instead thought to be a marker of other biological influences, including hormonal and growth factors, cellular divisions, and nutrition during formative years, that may contribute to cancer risk over the life course of an individual.

The reported positive associations between height and multiple subtypes of NHL suggest the existence of common biological pathways, possibly mediated by genetic factors. Height is a highly polygenic trait and estimated to be 70–80% heritable based on twin studies (17–19). Genome-wide association studies (GWAS) have identified hundreds of common variants associated with height to date, which explain approximately 16% of the variance of height (20), with additional rare variants also contributing to its variance (21). A deeper understanding of how height is associated with NHL risk may provide insight into underlying biological mechanisms leading to lymphoma.

In this study, we used previously established height-related genetic variants to construct polygenic risk scores (PRS) for height in order to examine the associations between genetically-inferred height and the risk of four common NHL subtypes: DLBCL, FL, CLL, and MZL.

## Methods

We used data from eight GWAS of NHL subtypes in populations of European ancestry (Supplementary Table 1), the details of which have been reported previous for DLBCL (22), FL (23), CLL (24), and MZL (25). The largest GWAS, the National Cancer Institute (NCI) NHL GWAS, consisted of cases and controls of European ancestry from 22 studies of NHL, including nine prospective cohort studies, eight population-based case-control studies, and five hospital or clinic-based case-control or case-series studies. The other seven GWAS consisted of two population-based case-control studies, one combined clinic- and population-based case-control study, one clinic-based case-control study with additional registry cases, one case series with population-based controls, one randomized clinical trial with population-based controls, and one case-control study enriched for familial cases (Supplementary Table 1). The 29 epidemiologic studies contributing to these GWAS were conducted in the North America, Australia, and Europe. In total, genotype data were available for 3,100 CLL cases, 3,857 DLBCL cases, 2,847 FL cases, and 825 MZL cases, and 9,505 controls. All studies obtained informed consent from participants and were approved by their appropriate Institutional Review Boards.

Genotyping was performed on commercially available Illumina and Affymetrix platforms (Supplementary Table 1). Standard quality control and filtering were applied to each GWAS, and each GWAS was imputed separately. Imputation was performed using IMPUTE2 (26) and the 1,000 Genomes Project version 2, February 2012 release (27) as the reference panel. As reported previously (22–25), quantile-quantile plots of the results showed no substantial evidence of inflation.

Previous studies have reported 848 independent, autosomal SNPs to be associated with height in primarily in European populations (20, 21). Wood et al. (20) identified 697 independent SNPs associated with height based on an approximate conditional analysis, taking the linkage disequilibrium among SNPs into account. Marouli et al. (21) identified an additional 113 novel autosomal loci, which were > 1 Mb from the loci reported by Wood et al. and 38 SNPs near established loci that were shown to be independent of previously reported SNPs based on conditional analyses. We constructed weighted polygenic risk scores (PRS) using 833 of these previously reported SNPs, which were available in our datasets with information scores > 0.3 and *p*-values for Hardy-Weinberg equilibrium >1 × 10^−5^. To generate the PRS, the height-increasing allelic dosage for each SNP was multiplied by the reported meta-analysis beta coefficient (20, 21), as shown below, where *w*_*j*_ is the weight or beta coefficient for the *j*th SNP derived from the literature and x_ij_ is the allelic dosage of the jth SNP.

$${\text{PRS}}_{\text{i}}=\sum_{\text{j = 1}}^{\text{k}}{\text{w}}_{\text{j}}{\text{x}}_{\text{ij}}$$

As previous studies have shown etiologic heterogeneity among NHL subtypes (1), we analyzed each NHL subtype separately. For each GWAS, we performed logistic regression with the PRS, which was normally distributed, modeled as a continuous variable. Models were adjusted for sex, age, and statistically significant principal components for population stratification (*p* < 0.05) at the GWAS level. Since all controls in the UCSF1/NHS GWAS were female, models for this GWAS were adjusted for age and significant principal components of ancestry only. In a sensitivity analysis, we also adjusted for genetically determined body mass index (BMI), using previously established loci. The regression analyses were performed in R using the *glm* function.

For each NHL subtype, GWAS-specific risk estimates were pooled using fixed-effect meta-analysis (28). Only one GWAS contributed to MZL, so no meta-analysis was performed for MZL. Between-GWAS heterogeneity was evaluated by the Cochran's Q test and quantified using the *I*^2^ metric (29). Meta-analyses were conducted using the package *metan* in Stata v15 (StataCorp, College Station, TX). The UCSF1/NHS GWAS was excluded from the sex-specific meta-analysis of FL because all controls were female. Between-sex heterogeneity was evaluated by performing a fixed-effect meta-analysis to obtain the *p*-value from the Cochran's Q-test and *I*^2^.

## Results

We found suggestive evidence for an association between taller genetically determined height and risk of CLL (OR = 1.08, 95% CI 1.00–1.17, *p* = 0.049), with the PRS modeled as a continuous variable (Table 1). Additional adjustment for BMI did not substantially alter the association (OR = 1.07, 95% CI 0.99–1.16). This association was stronger among females (OR = 1.15, 95% CI 1.01–1.31, *p* = 0.04) than among males (OR = 1.06, 95% CI 0.96–1.18, *p* = 0.26), but the between-sex heterogeneity was not statistically significant (*p* = 0.34). No significant heterogeneity among studies was observed for CLL among males and females combined (*I*^2^ = 0%, p_het_ = 0.69, Supplementary Table 2).

**Table 1:** Association between genetically predicted height and the risk of NHL by subtype.

	**No. cases/ No. controls**	**OR**	**95% CI**	***P***	*I*^2^^*^ (%)	**${\text{P}}_{\text{het}}^{*}$**
**CLL**						
Men + Women	3100/7666	**1.08**	**1.00–1.17**	**0.049**	0.0	0.690
Men	1794/5370	1.06	0.96–1.18	0.264	0.0	0.444
Women	1306/2296	**1.15**	**1.01–1.31**	**0.036**	0.0	0.732
**DLBCL**						
Men + Women	3587/7666	1.05	0.97–1.14	0.209	27.2	0.249
Men	1968/5260	1.02	0.92–1.13	0.731	2.1	0.382
Women	1889/2406	1.08	0.96–1.22	0.205	0.0	0.539
**FL**						
Men + Women	2847/8107	1.06	0.97–1.16	0.230	57.5	0.070
Men^†^	1276/5208	1.03	0.91–1.17	0.667	0.0	0.515
Women^†^	1452/2550	1.05	0.92–1.20	0.470	67.2	0.048
**MZL**						
Men + Women	825/6221	1.10	0.95–1.28	0.201		
Men	334/4527	1.19	0.96–1.49	0.116		
Women	491/1694	1.00	0.82–1.23	0.967		

* *Heterogeneity among GWAS. Only one GWAS contributed to MZL, so heterogeneity not evaluated for that subtype*.

† *Sex-specific FL analysis excludes UCSF1/NHS study*.

No association was observed between genetically predicted height and the risk of DLBCL (OR = 1.05, 95% CI 0.97–1.14), FL (OR = 1.06, 95% CI 0.97–1.16), or MZL (OR = 1.10, 95% CI 0.95–1.28) (Table 1). Adjustment for BMI did not alter these results (data not shown). For FL, there was some heterogeneity in the GWAS-specific risk estimates (*I*^2^ = 57.5%, p_het_ = 0.07, Supplementary Table 2), attributable primarily to the inclusion of the SCALE study. Removal of SCALE from the meta-analysis for FL risk slightly strengthened the combined association, but the resulting association did not reach statistical significance (OR = 1.09, 95% CI 0.99–1.20, *p* = 0.08). With regards to sex-specific analyses for these three subtypes, no significant associations were seen among men or women, with risk estimates similar to those observed for both sexes combined.

## Discussion

In this first examination of genetically inferred height and NHL risk, we observed a borderline positive association between genetically inferred taller height and the risk of CLL. This finding is consistent with observations from epidemiologic studies examining height and CLL risk, which generally have reported positive or insignificant associations (2, 3, 5, 11). The prospective UK Million Women Study, with 920 CLL cases, found an increased risk of CLL with increasing height (2). Although the Netherlands Cohort Study observed an overall association between taller adult height and risk of NHL overall, it did not observe a significantly elevated risk of CLL with 165 CLL cases (5). A previous pooled analysis within the InterLymph Consortium found an overall positive association between reported height and risk of CLL with a modest magnitude of association (OR per 10-cm increase = 1.10, 95% CI = 1.02–1.19) (11), which is comparable to the association with a one-unit increase in the PRS from the present study, but without a suggestion of heterogeneity by sex. Collectively, the present and published data provide evidence for a positive association between adult height and risk of CLL, although additional studies are needed to clarify possible sex differences.

No evidence of association was observed between genetically determined height and the risk of DLBCL, FL, nor MZL. Although the findings are not consistent (3, 8), some epidemiologic studies have reported positive findings for common NHL subtypes and height (2, 30). The UK Million Women Study reported a positive association with height for FL, DLBCL, and NHL overall (2). A large pooled analysis within the InterLymph Consortium found that greater adult height was positively associated FL for males, but not females (12). Consistent with our results, no significant associations were reported between adult height and MZL or DLBCL in the InterLymph pooled analysis (13, 31); however, an increased risk of DLBCL was observed for both sexes combined comparing the highest with the lowest quartile of usual adult height in a minimally adjusted model (31). It should be noted that some of the cases and controls included in our analysis were also included in the InterLymph pooled analyses of reported height and NHL risk (32); however, our study included 2,550 cases and 4,330 controls from prospective studies as well, which were not part of the pooled InterLymph analyses (Supplementary Table 1).

Despite the suggestive associations in the literature for self-reported or measured height and FL and DLBCL risk, there are several possible reasons why our study of genetically determined adult height did not identify statistically significant associations. A recent methodologic paper on the use of polygenic risk scores demonstrated that null results are often explained by inadequate sample size (33). Even though our study utilized data from the largest GWAS of NHL to date, it is a possible that our study was underpowered to detect an association for these NHL subtypes. In addition, it is possible that the genetic component of height that is pleiotropically related to risk of these NHL subtypes is not well-captured by the current set of height-associated variants. The PRS for height constructed for the present analyses accounts for <20% of the heritable component of adult height (20, 21). A PRS incorporating a larger fraction of the heritable component of adult height would provide a more precise measure of the genetic contribution to height and allow for a better assessment of the association with NHL risk. As more of the genetic component of height becomes identified, it may be possible to create a better PRS that accounts for a larger percentage of height variation. Finally, it is also possible that it is the non-genetic component of height, such as childhood nutrition, or a factor correlated with height that is responsible for the reported epidemiologic associations between adult stature and risk of NHL.

Among the noteworthy limitations of our study is the fact that our participants were from populations of European descent, limiting the generalizability of results. However, this restriction to Europeans also has the benefit of minimizing bias from population stratification, which is an important concern with studies of height. We did not adjust for multiple testing, because previous studies have shown individual NHL subtypes to have distinct etiologies (1). However, it is possible that the finding we observed with CLL may be a false-positive result, and additional studies are needed to replicate our findings. We were unable to perform a true Mendelian randomization analysis due to lack of data on height from many studies, but we were able to combine multiple GWAS of NHL by meta-analysis to achieve sample sizes of several thousand cases for three of the four subtypes. This approach further enabled a direct assessment of between-study and between-sex heterogeneity of the observed associations. Future studies with even larger sample sizes may be able to evaluate the shared genetic correlation with height using cross-trait LD Score regression.

In conclusion, we present the first polygenic risk score analysis examining the genetic contribution of attained adult height on the risk for the four most common NHL subtypes. Although we did not observe statistically significant associations with DLBCL, FL, or MZL, we observed evidence of a positive association between genetically-inferred height and CLL risk, lending more support to previous observational studies. Additional studies are warranted to explore the association between genetically-inferred height and risk of other NHL subtypes, particularly those that have been reported to be associated with self-reported or measured height in epidemiologic studies (34, 35). Additional insight could be gained from studies that explore the relationship between environmental or endogenous factors that may be correlated with or determinants of height and NHL risk. As more knowledge is gained about the genetic architecture and determinants of height, a more informative PRS can be constructed to represent a larger fraction of the heritable component of adult height and underlying biological pathways. A re-examination of the associations of NHL subtypes with genetically-inferred height may be merited in the future as our understanding of height and its underlying biology grows.

## Data Availability Statement

The datasets generated for this study can be found in the dbGap phs000801.v2.p1 at https://www.ncbi.nlm.nih.gov/projects/gap/cgi-bin/study.cgi?study_id=phs000801.v2.p1.

## Ethics Statement

All studies involving human participants were reviewed and approved by their respective Institutional Review Boards. The patients/participants provided their written informed consent to participate in this study.

## Author Contributions

AMoo and SIB organized and designed the study. AMoo, EK, LRT, MJM, OP, AMon, NW, ZW, CFS, JRC, and SIB contributed to the design and execution of data analysis. AMoo and SIB wrote the first draft of the manuscript. EK, LRT, AMon, NW, CFS, SLS, RV, CV, YZ, KS, JS, VJ, GG, BL, AA, AN, PMB, NJC, GS, WC, HH, H-OA, DA, NB, YB, SB, PBo, PBr, AB-W, FC, JC, LC, DC, KC, GF, LF, SG, HG, MG, BG, TH, RJ, QL, ML, MMa, JM, MMe, LM, RM, TM, LMM, KN, KO, MPu, AP, VR, ER, SS, RS, MS, AS, LFT, RT, EW, SW, TZ, NR, BB, JRC, and SIB conducted the epidemiological studies involved this study. All authors contributed to the writing of the manuscript.

### Conflict of Interest

The authors declare that the research was conducted in the absence of any commercial or financial relationships that could be construed as a potential conflict of interest.

## References

[1] Morton LM Slager SL Cerhan JR Wang SS Vajdic CM Skibola CF. Etiologic heterogeneity among non-Hodgkin lymphoma subtypes: the InterLymph Non-Hodgkin Lymphoma Subtypes Project. J Natl Cancer Inst Monogr. (2014) 2014:130–44. 10.1093/jncimonographs/lgu013 25174034 PMC4155467

[2] Murphy F Kroll ME Pirie K Reeves G Green J Beral V. Body size in relation to incidence of subtypes of haematological malignancy in the prospective Million Women Study. Br J Cancer. (2013) 108:2390–8. 10.1038/bjc.2013.159 23640394 PMC3681016

[3] Patel AV Diver WR Teras LR Birmann BM Gapstur SM. Body mass index, height and risk of lymphoid neoplasms in a large United States cohort. Leuk Lymphoma. (2013) 54:1221–7. 10.3109/10428194.2012.742523 23098244

[4] Kabat GC Kim MY Jean Wactawski W Bea JW Edlefsen KL Adams-Campbell LL. Anthropometric factors, physical activity, and risk of non-Hodgkin's lymphoma in the Women's Health Initiative. Cancer Epidemiol. (2012) 36:52–9. 10.1016/j.canep.2011.05.014 21816698 PMC6086363

[5] Pylypchuk RD Schouten LJ Goldbohm RA Schouten HC van den Brandt PA. Body mass index, height, and risk of lymphatic malignancies: a prospective cohort study. Am J Epidemiol. (2009) 170:297–307. 10.1093/aje/kwp123 19478235

[6] Britton JA Khan AE Rohrmann S Becker N Linseisen J Nieters A. Anthropometric characteristics and non-Hodgkin's lymphoma and multiple myeloma risk in the European Prospective Investigation into Cancer and Nutrition (EPIC). Haematologica. (2008) 93:1666–77. 10.3324/haematol.13078 18835833

[7] Lim U Morton LM Subar AF Baris D Stolzenberg-Solomon R Leitzmann M. Alcohol, smoking, and body size in relation to incident Hodgkin's and non-Hodgkin's lymphoma risk. Am J Epidemiol. (2007) 166:697–708. 10.1093/aje/kwm122 17596266

[8] Cerhan JR Janney CA Vachon CM Habermann TM Kay NE Potter JD. Anthropometric characteristics, physical activity, and risk of non-Hodgkin's lymphoma subtypes and B-cell chronic lymphocytic leukemia: a prospective study. Am J Epidemiol. (2002) 156:527–35. 10.1093/aje/kwf082 12226000

[9] Troy JD Hartge P Weissfeld JL Oken MM Colditz GA Mechanic LE. Associations between anthropometry, cigarette smoking, alcohol consumption, and non-Hodgkin lymphoma in the Prostate, Lung, Colorectal, and Ovarian Cancer Screening Trial. Am J Epidemiol. (2010) 171:1270–81. 10.1093/aje/kwq085 20494998 PMC2915494

[10] Leiba M Leiba A Keinan-Boker L Avigdor A Derazne E Levine H. Adolescent weight and height are predictors of specific non-Hodgkin lymphoma subtypes among a cohort of 2,352,988 individuals aged 16 to 19 years. Cancer. (2016) 122:1068–77. 10.1002/cncr.29792 26900677

[11] Slager SL Benavente Y Blair A Vermeulen R Cerhan JR Costantini AS. Medical history, lifestyle, family history, and occupational risk factors for chronic lymphocytic leukemia/small lymphocytic lymphoma: the InterLymph Non-Hodgkin Lymphoma Subtypes Project. J Natl Cancer Inst Monogr. (2014) 2014:41–51. 10.1093/jncimonographs/lgu001 25174025 PMC4155456

[12] Linet MS Vajdic CM Morton LM de Roos AJ Skibola CF Boffetta P. Medical history, lifestyle, family history, and occupational risk factors for follicular lymphoma: the InterLymph Non-Hodgkin Lymphoma Subtypes Project. J Natl Cancer Inst Monogr. (2014) 2014:26–40. 10.1093/jncimonographs/lgu006 25174024 PMC4155461

[13] Bracci PM Benavente Y Turner JJ Paltiel O Slager SL Vajdic CM. Medical history, lifestyle, family history, and occupational risk factors for marginal zone lymphoma: the InterLymph Non-Hodgkin Lymphoma Subtypes Project. J Natl Cancer Inst Monogr. (2014) 2014:52–65. 10.1093/jncimonographs/lgu011 25174026 PMC4207869

[14] Thrift AP Gong J Peters U Chang-Claude J Rudolph A Slattery ML. Mendelian randomization study of height and risk of colorectal cancer. Int J Epidemiol. (2015) 44:662–72. 10.1093/ije/dyv082 25997436 PMC4481609

[15] Zhang B Shu XO Delahanty RJ Zeng C Michailidou K Bolla MK. Height and breast cancer risk: evidence from prospective studies and mendelian randomization. J Natl Cancer Inst. (2015) 107:djv219. 10.1093/jnci/djv219 26296642 PMC4643630

[16] Khankari NK Shu XO Wen W Kraft P Lindstrom S Peters U. Association between adult height and risk of colorectal, lung, and prostate cancer: results from meta-analyses of prospective studies and Mendelian randomization analyses. PLoS Med. (2016) 13:e1002118. 10.1371/journal.pmed.1002118 27598322 PMC5012582

[17] Silventoinen K Sammalisto S Perola M Boomsma DI Cornes BK Davis C. Heritability of adult body height: a comparative study of twin cohorts in eight contries. Twin Res. (2003) 6:399–408. 10.1375/136905203770326402 14624724

[18] Visscher PM Medland SE Ferreira MA Morley KI Zhu G Cornes BK. Assumption-free estimation of heritability from genome-wide identity-by-descent sharing between full siblings. PLoS Genet. (2006) 2:e41. 10.1371/journal.pgen.0020041 16565746 PMC1413498

[19] Jelenkovic A Sund R Hur YM Yokoyama Y Hjelmborg JV Moller S. Genetic and environmental influences on height from infancy to early adulthood: an individual-based pooled analysis of 45 twin cohorts. Sci Rep. (2016) 6:28496. 10.1038/srep28496 27333805 PMC4917845

[20] Wood AR Esko T Yang J Vedantam S Pers TH Gustafsson S. Defining the role of common variation in the genomic and biological architecture of adult human height. Nat Genet. (2014) 46:1173–86. 10.1038/ng.3097 25282103 PMC4250049

[21] Marouli E Graff M Medina-Gomez C Lo KS Wood AR Kjaer TR. Rare and low-frequency coding variants alter human adult height. Nature. (2017) 542:186–90. 10.1038/nature21039 28146470 PMC5302847

[22] Cerhan JR Berndt SI Vijai J Ghesquieres H McKay J Wang SS. Genome-wide association study identifies multiple susceptibility loci for diffuse large B cell lymphoma. Nat Genet. (2014) 46:1233–8. 10.1158/1538-7445.AM2014-LB-272 25261932 PMC4213349

[23] Skibola CF Berndt SI Vijai J Conde L Wang Z Yeager M. Genome-wide association study identifies five susceptibility loci for follicular lymphoma outside the HLA region. Am J Hum Genet. (2014) 95:462–71. 10.1016/j.ajhg.2014.09.004 25279986 PMC4185120

[24] Berndt SI Skibola CF Joseph V Camp NJ Nieters A Wang Z. Genome-wide association study identifies multiple risk loci for chronic lymphocytic leukemia. Nat Genet. (2013) 45:868–76. 10.1158/1538-7445.AM2013-LB-23 23770605 PMC3729927

[25] Vijai J Wang Z Berndt SI Skibola CF Slager SL de Sanjose S. A genome-wide association study of marginal zone lymphoma shows association to the HLA region. Nat Commun. (2015) 6:5751. 10.1038/ncomms6751 25569183 PMC4287989

[26] Howie BN Donnelly P Marchini J. A flexible and accurate genotype imputation method for the next generation of genome-wide association studies. PLoS Genet. (2009) 5:e1000529. 10.1371/journal.pgen.1000529 19543373 PMC2689936

[27] Genomes Project C Abecasis GR Altshuler D Auton A Brooks LD Durbin RM. A map of human genome variation from population-scale sequencing. Nature. (2010) 467:1061–73. 10.1038/nature09534 20981092 PMC3042601

[28] Panagiotou OA Willer CJ Hirschhorn JN Ioannidis JP. The power of meta-analysis in genome-wide association studies. Annu Rev Genomics Hum Genet. (2013) 14:441–65. 10.1146/annurev-genom-091212-153520 23724904 PMC4040957

[29] Higgins JP Thompson SG. Quantifying heterogeneity in a meta-analysis. Stat Med. (2002) 21:1539–58. 10.1002/sim.1186 12111919

[30] Cerhan JR Bernstein L Severson RK Davis S Colt JS Blair A. Anthropometrics, physical activity, related medical conditions, and the risk of non-hodgkin lymphoma. Cancer Causes Control. (2005) 16:1203–14. 10.1007/s10552-005-0358-7 16215871

[31] Cerhan JR Kricker A Paltiel O Flowers CR Wang SS Monnereau A. Medical history, lifestyle, family history, and occupational risk factors for diffuse large B-cell lymphoma: the InterLymph Non-Hodgkin Lymphoma Subtypes Project. J Natl Cancer Inst Monogr. (2014) 2014:15–25. 10.1093/jncimonographs/lgu010 25174023 PMC4155465

[32] Morton LM Sampson JN Cerhan JR Turner JJ Vajdic CM Wang SS. Rationale and design of the international lymphoma epidemiology consortium (InterLymph) non-hodgkin lymphoma subtypes project. J Natl Cancer Inst Monogr. (2014) 2014:1–14. 10.1093/jncimonographs/lgu005 25174022 PMC4155460

[33] Dudbridge F. Power and predictive accuracy of polygenic risk scores. PLoS Genet. (2013) 9:e1003348. 10.1371/journal.pgen.1003348 23555274 PMC3605113

[34] Mbulaiteye SM Morton LM Sampson JN Chang ET Costas L de Sanjose S. Medical history, lifestyle, family history, and occupational risk factors for sporadic Burkitt lymphoma/leukemia: the Interlymph Non-Hodgkin Lymphoma Subtypes Project. J Natl Cancer Inst Monogr. (2014) 2014:106–14. 10.1093/jncimonographs/lgu003 25174031 PMC4155458

[35] Monnereau A Slager SL Hughes AM Smith A Glimelius B Habermann TM. Medical history, lifestyle, and occupational risk factors for hairy cell leukemia: the InterLymph Non-Hodgkin Lymphoma Subtypes Project. J Natl Cancer Inst Monogr. (2014) 2014:115–24. 10.1093/jncimonographs/lgu004 25174032 PMC4155459

