# Supplementary material for: Genetically Determined Height and Risk of Non-hodgkin Lymphoma
Source: Front Oncol. 2020 Jan 28;9:1539. doi: 10.3389/fonc.2019.01539 (PMC6999122; doi:10.3389/fonc.2019.01539)
Supplement: Supplementary file 1 [file Data_Sheet_1.docx]

**Supplementary Table 2. Individual GWAS results for association between genetically predicted height and NHL risk by subtype**

| **Study** | **No. of cases/ No. of controls** | **OR** | **95% CI** | **P** |
| --- | --- | --- | --- | --- |
| **Chronic lymphocytic leukemia (CLL)** | | | | |
| *Men+Women* |  |  |  |  |
| NCI | 2179/6221 | 1.06 | (0.97-1.16) | 0.23 |
| GEC | 387/294 | 1.19 | (0.87-1.62) | 0.28 |
| UCSF2 | 213/746 | 1.16 | (0.87-1.54) | 0.31 |
| Utah | 321/405 | 1.22 | (0.91-1.63) | 0.19 |
| **Combined** | **3100/7666** | **1.08** | **(1.00-1.17)** | **0.05** |
| *Men* |  |  |  |  |
| NCI | 1214/4527 | 1.02 | (0.91-1.15) | 0.72 |
| GEC | 252/186 | 1.41 | (0.94-2.12) | 0.1 |
| UCSF2 | 136/430 | 1.10 | (0.77-1.57) | 0.61 |
| Utah | 192/227 | 1.19 | (0.81-1.75) | 0.38 |
| **Combined** | **1794/5370** | **1.06** | **0.96-1.18** | **0.26** |
| *Women* |  |  |  |  |
| NCI | 965/1694 | 1.15 | (0.99-1.33) | 0.07 |
| GEC | 135/108 | 0.92 | (0.57-1.5) | 0.74 |
| UCSF2 | 77/316 | 1.28 | (0.81-2.03) | 0.29 |
| Utah | 129/178 | 1.31 | (0.82-2.08) | 0.26 |
| **Combined** | **1306/2296** | **1.15** | **(1.01-1.31)** | **0.04** |
| **Diffuse Large B-cell Lymphoma (DLBCL)** | | | | |
| Men+Women |  |  |  |  |
| NCI | 2661/6221 | 1.05 | (0.96-1.16) | 0.27 |
| GELA/EPIC | 549/525 | 0.86 | (0.68-1.10) | 0.23 |
| MAYO | 393/172 | 1.08 | (0.75-1.56) | 0.68 |
| UCSF2 | 254/748 | 1.20 | (0.97-1.47) | 0.09 |
| **Combined** | **3587/7666** | **1.05** | **(0.97-1.14)** | **0.21** |
| *Men* |  |  |  |  |
| NCI | 1306/4527 | 1.04 | (0.92-1.17) | 0.57 |
| GELA/EPIC | 313/236 | 0.76 | (0.53-1.08) | 0.13 |
| MAYO | 207/66 | 1.1 | (0.64-1.92) | 0.72 |
| UCSF2 | 142/431 | 1.1 | (0.83-1.45) | 0.52 |
| **Combined** | **1968/5260** | **1.02** | **(0.92-1.13)** | **0.73** |
| *Women* |  |  |  |  |
| NCI | 1355/1694 | 1.05 | (0.91-1.21) | 0.5 |
| GELA/EPIC | 236/289 | 1.01 | (0.72-1.42) | 0.96 |
| MAYO | 186/106 | 1.01 | (0.61-1.66) | 0.98 |
| UCSF2 | 112/317 | 1.33 | (0.98-1.82) | 0.07 |
| **Combined** | **1889/2406** | **1.08** | **(0.96-1.22)** | **0.21** |
| **Follicular Lymphoma (FL)** | | | | |
| *Men+Women* |  |  |  |  |
| NCI | 2142/6221 | 1.05 | (0.95-1.17) | 0.33 |
| SCALE | 376/791 | 0.84 | (0.65-1.09) | 0.19 |
| UCSF2 | 119/349 | 1.42 | (1.04-1.95) | 0.03 |
| UCSF1/NHS | 210/746 | 1.27 | (0.81-2.00) | 0.30 |
| **Combined** | **2847/8107** | **1.06** | **(0.97-1.16)** | **0.23** |
| *Men^*^* |  |  |  |  |
| NCI | 980/4527 | 1.04 | (0.9-1.2) | 0.62 |
| SCALE | 186/251 | 0.86 | (0.59-1.26) | 0.44 |
| UCSF2 | 110/430 | 1.20 | (0.78-1.85) | 0.41 |
| **Combined** | **1276/5208** | **1.03** | **(0.91-1.17)** | **0.67** |
| *Women^*^* |  |  |  |  |
| NCI | 1162/1694 | 1.04 | (0.9-1.21) | 0.57 |
| SCALE | 190/540 | 0.83 | (0.59-1.17) | 0.28 |
| UCSF2 | 100/316 | 1.71 | (1.07-2.72) | 0.02 |
| **Combined** | **1452/2550** | **1.05** | **(0.92-1.20)** | **0.47** |
| ^*^Sex-specific combined results exclude UCSF1/NHS | | |  |  |
